# Supplementary material for: A Critical View of the Application of the APEX Software (Aqueous Photochemistry of Environmentally-Occurring Xenobiotics) to Predict Photoreaction Kinetics in Surface Freshwaters
Source: Molecules. 2019 Dec 18;25(1):9. doi: 10.3390/molecules25010009 (PMC7017383; doi:10.3390/molecules25010009)
Supplement: Supplementary file 1 [file molecules-25-00009-s001.zip › 3.2.4_gcc-4.4.0.zip!/3.2.4_gcc-4.4.0/3.2.4_gcc-4.4.0/doc/octave/html/interpreter/Polynomial-Manipulations.html]

Polynomial Manipulations - Untitled


Next: Interpolation,
Previous: Sets,
Up: Top


---

## 27 Polynomial Manipulations

In Octave, a polynomial is represented by its coefficients (arranged
in descending order). For example, a vector c of length
N+1 corresponds to the following polynomial of order
N

```
     p(x) = c(1) x^N + ... + c(N) x + c(N+1).
```

- Evaluating Polynomials- Finding Roots- Products of Polynomials- Derivatives and Integrals- Polynomial Interpolation- Miscellaneous Functions
